# Endometriosis and endometrial cancer: A propensity score-adjusted real-world data study

**DOI:** 10.1016/j.isci.2024.109680

**Published:** 2024-04-06

**Authors:** Alberto Farolfi, Nicola Gentili, Sara Testoni, Francesca Rusconi, Ilaria Massa, Valentina Danesi, Amelia Altavilla, Maria C. Cursano, Giorgia Gurioli, Salvatore L. Burgio, Gema Hernandez Ibarburu, Ugo De Giorgi

**Affiliations:** 1 Department of Medical Oncology, IRCCS Istituto Romagnolo per lo Studio dei Tumori (IRST) “Dino Amadori”, Meldola, Italy; 2 Outcome Research, IRCCS Istituto Romagnolo per lo studio dei Tumori (IRST) “Dino Amadori”, Meldola, ItalyW; 3 Biostatistics and Clinical Trials Unit, IRCCS Istituto Romagnolo per lo Studio dei Tumori (IRST) “Dino Amadori”, Meldola, Italy; 4 TriNetX, LLC, Cambridge, MA, USA; 5 Biosciences Laboratory, IRCCS Istituto Romagnolo per lo Studio dei Tumori (IRST) “Dino Amadori”, Meldola, Italy

**Keywords:** Health sciences, Public health, Female reproductive endocrinology, Cancer

## Abstract

Endometriosis is a benign condition characterized by the presence of ectopic endometrial tissue. Our study investigated the effect of endometriosis on the risk of endometrial cancer (EC) and the prognosis of endometriosis-associated EC. In our study, 197,196 patients with endometriosis and without a previous diagnosis of EC were compared with 6,455,556 females encountering health services for examinations, with body mass index (BMI) data, and without endometriosis or EC. A propensity score generated 197,141 matched pairs. In the endometriosis cohort, 875 cases of EC were seen, whereas 558 were in the control group: the hazard ratio (HR) was 1.56 (95% CI 1.40–1.73, *p* < 0.001). Women with endometriosis were more likely to develop invasive endometrioid (*p* = 0.005) and clear cell (*p* < 0.001) EC. There was no difference in overall survival between endometriosis-associated EC and EC without endometriosis. Our epidemiological findings were consistent with the evidence of an association between endometriosis and EC.

## Highlights

• 197,141 matched pairs of patients with or without endometriosis were analyzed
• Endometriosis demonstrated to be a risk factor for endometrial cancer (EC)
• Endometrioid and clear cell EC histologies were associated with endometriosis
• The prognosis of EC was not affected by a previous diagnosis of endometriosis

## Graphical abstract

**Figure:**
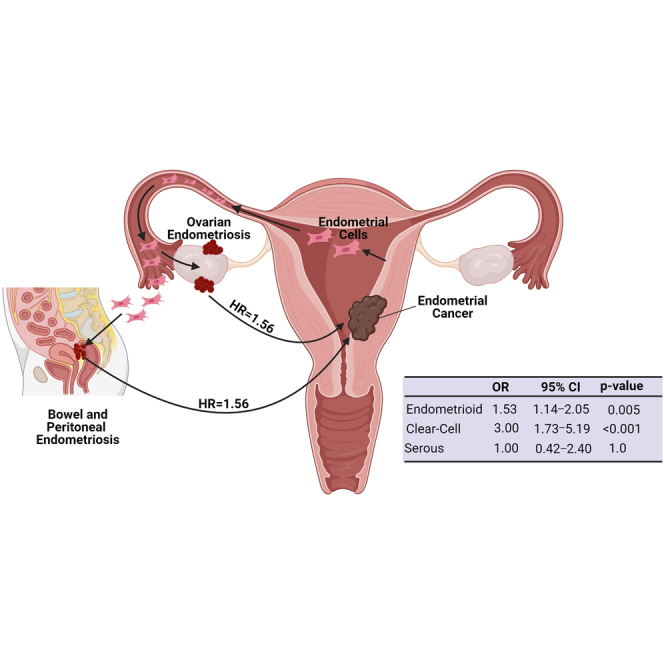

## Introduction

Endometriosis is a relatively common disease characterized by abnormal implantation of the endometrial glands and stroma at ectopic sites outside the uterus, resulting in chronic inflammation, pain, and infertility.^1^ Endometriosis foci are typically found on the surface of ovaries and pelvic peritoneum. The etiology underlying endometriosis is controversial, but it is suggested that its development processes closely resemble those involved in cancer metastasis.^2^ Although the most accredited theory of its origin assumes that it develops following retrograde menstruation.^3^^,^^4^ Other causes could be implicated, including genetic and environmental factors, alteration of the immune system, and ectopic differentiation of the mesenchymal stem cells.^5^

The estimated prevalence of endometriosis in the general population is about 4%.^6^ However, the disease is much more common, occurring in approximately 10% of premenopausal women,^7^ and is present in up to 50% of women with chronic pelvic pain and 30–50% of women with infertility.^8^

Although endometriosis is considered a benign disorder from both a clinical and a histopathological perspective, well-known cancer-associated somatic mutations were found in the glandular epithelium of some deep-infiltrating endometriosis lesions.^9^ Indeed, ovarian endometriosis was found to be associated with an increased risk of epithelial ovarian cancer.^10^ A pooled analysis of a case-control study demonstrated that self-reported endometriosis was associated with specific histological subtypes of ovarian cancer, and endometriosis should be thought of as a precursor lesion for clear-cell, endometrioid ovarian cancers, and low-grade serous ovarian cancers.^11^

How endometriosis can become malignant is still a matter of debate. Although endometriosis (ovarian and extraovarian endometriotic lesions) carries somatic mutations in cancer driver genes,^9^^,^^12^ it has been shown that the same somatic mutations are also commonly found in “normal” endometrial tissue.^13^ These observations have raised the question of whether women with endometriosis are at a higher risk of developing endometrial cancer (EC). A retrospective case-control study conducted in Taiwan demonstrated that 20,510 patients with endometriosis had a significantly higher risk of EC (hazard ratio (HR) = 2.92; 95% CI = 2.12–4.03).^14^ However, many meta-analyses have reached different conclusions: one found a slight but statistically significant increased risk of EC (relative risk (RR) = 1.66; 95% CI 1.15–2.41),^15^ whereas others observed a non-statically significant relationship.^16^^,^^17^

Currently, there is limited knowledge about the pathophysiology of endometriosis; having a clear answer to its link to EC has important public health implications for women in terms of cancer screening and prevention and for clinicians in terms of the long-term management of women with endometriosis. In this context, real-world data (RWD) could provide real-world evidence (RWE)^18^ to assess if endometriosis is a risk factor for EC and evaluate the prognosis of endometriosis-associated EC.

## Results

### Association between endometriosis and endometrial cancer diagnosis

**Figure 1:**
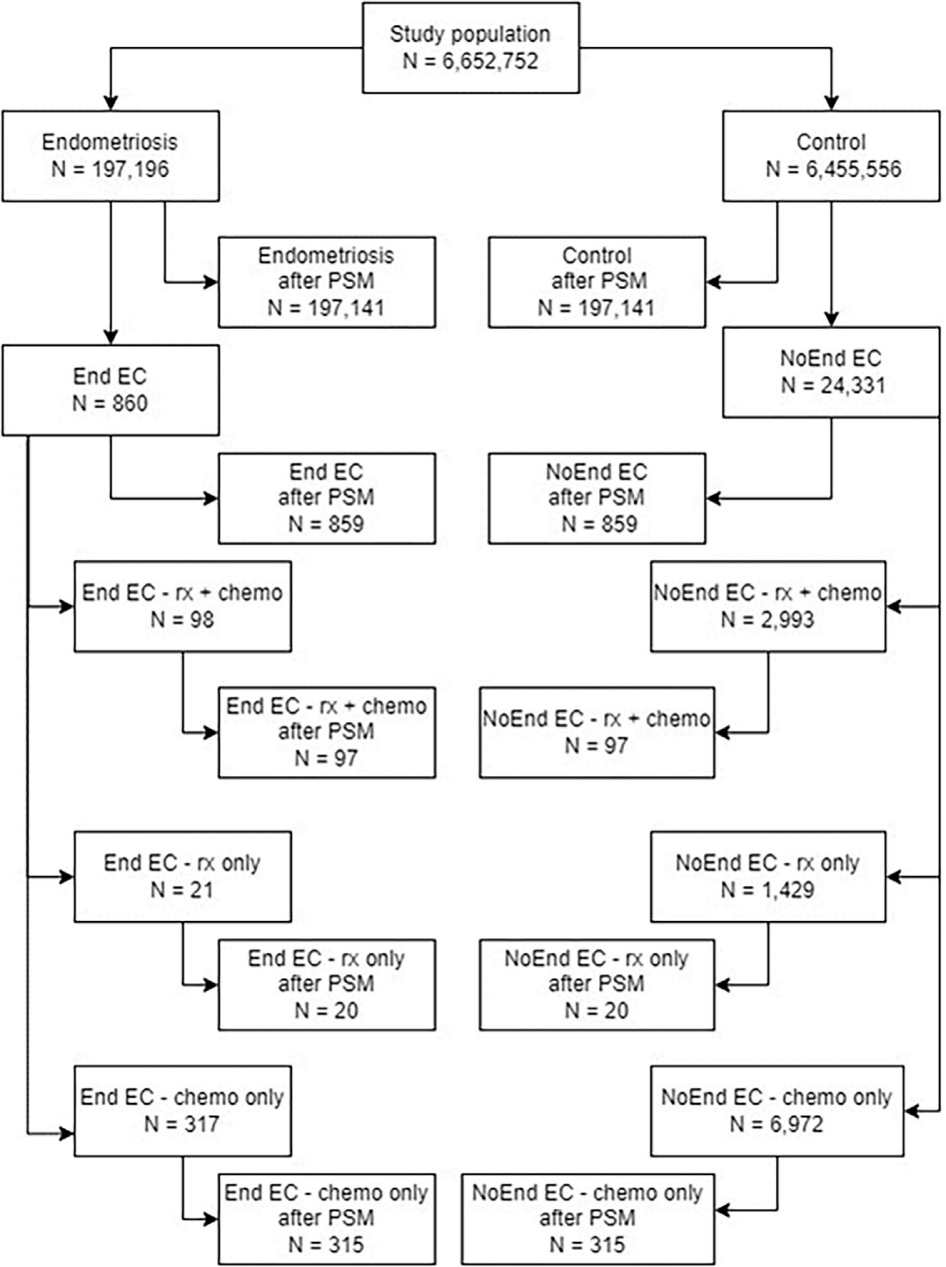
Patient flow chart Flow chart for the identification of cohorts analyzed.

Before propensity score matching (PSM), the study population contained 197,196 endometriosis patients and 6,455,556 controls. After PSM, the study population counted 197,141 endometriosis patients and 197,141 controls. Table 1 lists the covariate differences between the two groups before and after matching. Before matching, significant imbalances between the two groups were observed concerning age, race, pelvic inflammatory disease, family history or genetic susceptibility to neoplasms, previous colorectal cancer, and body mass index (BMI) categories. Figure 1 shows the patient flow chart of the cohorts that were analyzed.

**Table 1:** Patient characteristics before and after propensity score matching

Covariate	Before matching			After matching		
	Endometriosis *N* = 197,196 (%)	Control *N* = 6,455,556 (%)	*d*^a^ (%)	Endometriosis *N* = 197,141 (%)	Control *N* = 197,141 (%)	*d*^a^ (%)
Age–Mean ± SD (years)	40.2 ± 11.1	47.0 ± 16.6	0.482	40.2 ± 11.1	40.1 ± 11.1	0.005

**Race**						

Caucasian	123,025 (62.4%)	4,236,835 (65.6%)	0.068	122,978 (62.4%)	122,892 (62.3%)	0.001
Black	20,780 (10.5%)	923,446 (14.3%)	0.114	20,778 (10.5%)	20,764 (10.5%)	<0.001
Asian	7,857 (4.0%)	242,728 (3.8%)	0.012	7,855 (4.0%)	7,823 (4.0%)	0.001

**Diagnosis**						

Inflammatory diseases of female pelvic organs	47,258 (24.0%)	257,284 (4.0%)	0.602	47,203 (23.9%)	47,145 (23.9%)	0.001
Family history of primary malignant neoplasm	10,803 (5.5%)	204,725 (3.2%)	0.114	10,759 (5.5%)	10,635 (5.4%)	0.003
Genetic susceptibility to other malignant neoplasm	416 (0.2%)	2,672 (0.0%)	0.048	361 (0.2%)	387 (0.2%)	0.003
Malignant neoplasm of colon	641 (0.3%)	17,447 (0.3%)	0.010	635 (0.3%)	613 (0.3%)	0.002
Malignant neoplasm of rectosigmoid junction	152 (0.1%)	3,752 (0.1%)	0.007	151 (0.1%)	147 (0.1%)	0.001
Malignant neoplasm of rectum	200 (0.1%)	6,365 (0.1%)	0.001	199 (0.1%)	187 (0.1%)	0.002
Malignant neoplasm of ovary	2,455 (1.2%)	13,233 (0.2%)	0.123	2,411 (1.2%)	3,292 (1.3%)	0.004

**Body mass index (kg/m**^**2**^**)**						

Mean ± SD	28.7 ± 7.0	28.8 ± 7.1	0.005	28.7 ± 7.0	28.8 ± 7.1	<0.001
0–18.5	2,814 (1.4%)	65,316 (1.0%)	0.038	2,810 (1.4%)	2,768 (1.4%)	0.002
18.5–25	25,213 (12.8%)	560,353 (8.7%)	0.133	25,199 (12.8%)	25,176 (12.8%)	<0.001
25–30	23,928 (12.1%)	510,911 (7.9%)	0.141	23,915 (12.1%)	23,797 (12.1%)	0.002
>30	26,089 (13.2%)	605,622 (9.4%)	3.1	26,077 (13.2%)	25,824 (13.1%)	0.004

a Standard difference.

PSM generated 197,141 matched pairs. In the endometriosis cohort (*N* = 197,141), 875 cases of EC were seen, whereas 558 were in the control group (*N* = 197,141), with an HR of 1.56 (95% CI 1.40–1.73, *p* < 0.001). The median time from endometriosis to EC diagnosis was 337 days (IQR 43–1261 days).

Women with endometriosis were more likely to develop invasive endometrioid (110 versus 72 EC patients, odds ratio (OR) = 1.53, 95%CI 1.14–2.06, *p* = 0.005) and clear-cell endometrial cancer (51 versus 17 EC patients, OR 3.0, 95% CI 1.73–5.2, *p* < 0.001) compared to women without this diagnosis. A history of endometriosis was not associated with invasive serous EC (10 EC cases in patients without endometriosis versus 10 EC cases in patients with endometriosis). There were no differences found in the EC stage between endometriosis and the control cohort for early stages I to III. In the endometriosis cohort, a reduction in the risk for stage IV EC was seen: OR 0.61 (95% CI 0.44–0.84, *p* = 0.002).

### Association between endometriosis and endometrial cancer prognosis

Before PSM, we identified 860 endometriosis-related EC patients and 24,331 non-endometriosis-related EC patients. After PSM, the study group counted 859 matched pairs (Figure 1). Before matching, significant imbalances were observed between the two groups compared to all categories. Table 2 lists the covariate differences between the two groups before and after matching.

**Table 2:** Endometriosis and Non-endometriosis related endometrial cancer patient characteristics before and after propensity score matching

Covariate	Before matching			After matching		
	Endometriosis-related EC *N* = 860 (%)	Non-endometriosis EC *N* = 24,331 (%)	*d*^a^ (%)	Endometriosis-related EC *N* = 859 (%)	Non-endometriosis EC *N* = 859 (%)	*d*^a^ (%)
Age–Mean ± SD (years)	52.1 ± 11.4	62.1 ± 11.4	0.881	52.1 ± 11.4	51.6 ± 11.8	0.041

**Race**						

White	580 (67.4%)	17,778 (73.1%)	0.123	579 (67.4%)	580 (67.5%)	0.002
Black	63 (7.3%)	2,758 (11.3%)	0.048	63 (7.3%)	60 (7.0%)	0.014
Asian	54 (6.3%)	941 (3.9%)	0.098	54 (6.3%)	62 (7.2%)	0.041

**Diagnosis**						

Inflammatory diseases of female pelvic organs	271 (31.5%)	2,378 (9.8%)	0.558	270 (31.4%)	255 (29.7%)	0.038
Family history of primary malignant neoplasm	153 (17.8%)	3,571 (14.7%)	0.085	153 (17.8%)	153 (17.8%)	<0.001
Genetic susceptibility to other malignant neoplasm	13 (1.5%)	196 (0.8%)	0.066	13 (1.5%)	10 (1.2%)	0.030
Malignant neoplasm of colon	24 (2.8%)	439 (1.8%)	0.034	24 (2.8%)	11 (1.3%)	0.107
Malignant neoplasm of rectosigmoid junction	10 (1.2%)	111 (0.5%)	0.079	10 (1.2%)	10 (1.2%)	<0.001
Malignant neoplasm of rectum	10 (1.2%)	118 (0.5%)	0.075	10 (1.2%)	10 (1.2%)	<0.001
Malignant neoplasm of ovary	163(19.0%)	1,582 (6.5%)	0.382	160 (18.6%)	144 (16.8%)	0.048

**Body mass index (kg/m**^**2**^**)**						

Mean ± SD	31.1 ± 8.4	32.7 ± 8.1	0.194	31.1 ± 8.4	31.7 ± 8.2	0.068
0–18.5	26 (3.0%)	536 (2.2%)	0.051	26 (3.0%)	25 (2.9%)	0.007
18.5–25	114 (13.3%)	2,106 (8.7%)	0.148	114 (13.3%)	113 (13.2%)	0.003
25–30	136 (15.8%)	2,924 (12.0%)	0.110	135 (15.7%)	139 (16.2%)	0.013
>30	197 (22.9%)	5,588 (23.0%)	0.001	196 (22.8%)	198 (23.1%)	0.006

a Standard difference.

**Figure 2:**
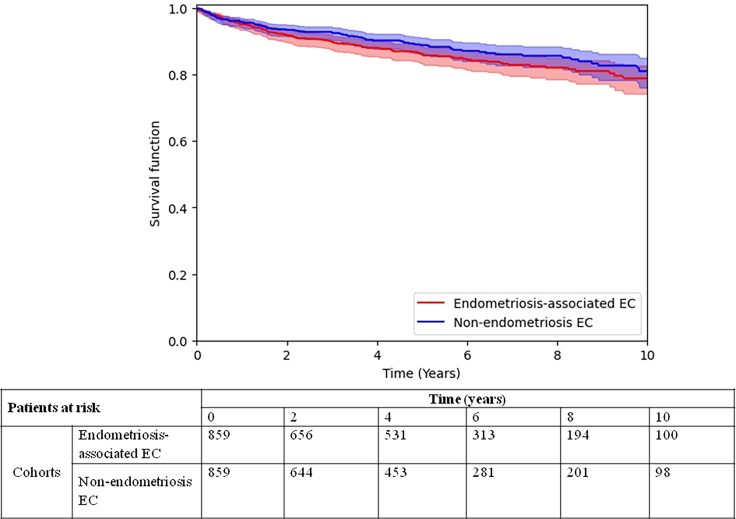
Overall survival Estimation of overall survival for patients with endometriosis-associated endometrial cancer (red line) and patients with endometrial cancer without endometriosis (blue line).

Among 859 endometriosis-associated EC patients, 118 (13.7%) patients died compared to 92 (10.7%) patients with EC without endometriosis: HR = 1.22 (95% CI 0.93–1.6, *p* = 0.702, Figure 2). Further analyses to evaluate the stage and histology role in the endometriosis-associated EC relationship showed no differences (data not shown).

PSM generated 97 matched pairs of EC patients treated with radiochemotherapy. There was no observed difference in overall survival (HR = 1.13, 95% CI 0.679–1.88, *p* = 0.506) between the group with endometriosis before the diagnosis of EC and the EC group without endometriosis. Similar results were seen in the 315 matched pairs of patients treated with chemotherapy alone (HR = 0.94, 95% CI 0.65–1.36, *p* = 0.342), or in the 20 matched patients who underwent radiotherapy alone (HR = 1.74, 95% CI 0.28–10.73, *p* = 0.108).

## Discussion

Although endometriosis has been associated with certain gynecological cancers, particularly ovarian cancer,^11^ the relationship with EC is complex and still the subject of ongoing research. Endometriosis and EC are distinct conditions, but they share similar risk factors, such as family history or nulliparity.^20^^,^^21^ Epidemiological studies have suggested a possible link between endometriosis and EC but with conflicting results.^14^^,^^15^^,^^16^^,^^17^

Our findings, obtained by RWD with a large sample size, suggest that a diagnosis of endometriosis is a risk factor for EC. The increased risk is consistent with all the histological subtypes apart from serous histology. Interestingly, the association between endometriosis and clear-cell EC, was highly significant, although the cases in our study were few, suggesting a common cancerogenesis with clear-cell epithelial ovarian cancer.^11^

The pathogenesis of endometriosis is complex and not yet fully defined, but it involves several well-supported molecular hallmarks, including genetic predisposition, estrogen dependence, progesterone resistance, and inflammation.^22^^,^^23^ The exact cause of endometriosis remains unclear, but many theories have been suggested, including retrograde menstruation, coelomic metaplasia, lymphovascular dissemination, and genetic and environmental factors.^4^^,^^22^ Endometriosis and EC share common etiological mechanisms, including estrogen stimulation and chronic inflammation.^16^ Additionally, genomic studies have demonstrated that cancer-related genomic alterations are present in some endometriotic lesions, supporting the hypothesis that endometriosis is the origin of ovarian endometrioid and clear cell carcinoma.^12^^,^^15^^,^^24^

Patients with deep-infiltrating endometriosis had lesions with 21% harboring somatic mutations in *ARID1A*, *PIK3CA*, *KRAS*, and *PPP2R1A*.^9^ Suda et al., detected activating mutations on *KRAS* and *PI3KCA* in 38% and 29% of endometriotic lesions, respectively, with a marked increase in the mutant allele frequency from normal epithelium to endometrial tissue, confirming these cancer-associated mutations as putative selective growth advantages leading to the development of endometriosis and a widespread distribution of the clone across the endometriotic lesions.^13^ A mouse model demonstrated that the subclonal activating mutations of *KRAS* can sustain endometriosis but are not sufficient for malignant transformation.^25^ However, the *KRAS* mutation associated with the *PTEN* loss-of-function mutation developed an endometrioid type of ovarian cancer in preclinical models.^26^ In another mouse model, it was observed that *ARID1A* and *PIK3CA* mutations cooperated to promote the growth of clear-cell ovarian cancer.^27^ It was demonstrated that papillary proliferation of endometrium, complex endometrial hyperplasia, and EC share common mutations, in which *KRAS* might be a driver,^28^ and mutations in *PTEN*, *PIK3CA*, and/or *ARID1A* might be necessary for furthering cancerogenesis.

In our study, we observed no differences in terms of prognosis between EC associated with endometriosis and sporadic EC, regardless of the treatment received. Our observation found a reduced rate of stage IV EC in the endometriosis cohort, underlining that this could be associated with the increased number of gynecological visits endometriosis patients experience during their clinical history.

This observation contrasts with the better survival rate seen in patients with EC and coexisting adenomyosis, which is defined as the presence of ectopic endometrium within the myometrium.^29^ Because adenomyosis, endometriosis, and cancer share a common pathogenic mechanism (cancer originating from adenomyosis is infrequent, especially among older individuals with only a 1% transformation rate),^30^ further studies are needed to better characterize EC associated with endometriosis. We would like to molecularly characterize EC to identify possible differences between sporadic cases and tumors arising after a diagnosis of endometriosis.

### Limitations of the study

In this context, our study presents some limitations that need to be discussed. The main limitation of our analysis is the absence of molecular data. Although the mismatch repair status was unavailable in the global collaborative network, we used the family history of neoplasm and a diagnosis of colorectal cancer as an indicator of Lynch syndrome.

There is another limitation that is inherent in the use of RWD. The identification of patients with endometriosis relies on diagnostic data recorded according to the International Classification of Diseases, Tenth Revision (ICD-10). It is worth noting that many healthcare institutions primarily code for severe cases of endometriosis for which surgical intervention is often required, potentially leading to a bias wherein patients with milder forms of the disease (diagnosed via imaging modalities), may not be adequately captured.^31^ However, this analysis is more reliable than self-reported diagnosis. This, in association with the large sample size, produces results that are very consistent since we were able to consider and control a wide range of potential confounders.

## STAR★Methods

### Key resources table

**Table:** 

REAGENT or RESOURCE	SOURCE	IDENTIFIER
**Software and algorithms**		

TriNetX platform	TriNetX, LLC	https://live.trinetx.com/

### Resource availability

#### Lead contact

Further information and resource requests should be directed to the lead contact: Dr Alberto Farolfi (alberto.farolfi@irst.emr.it).

#### Materials availability

This study did not use or generate reagents.

#### Data and code availability

• The patients’ data reported in this study cannot be deposited in a public repository because third-party restrictions apply to the availability of these data. The data were used under license for this study with restrictions that do not allow for the data to be redistributed or made publicly available. To request access, contact TriNetX, LLC (data access may require a data sharing agreement and may incur data access fees.).
• This paper does not report the original code.
• Any additional information required to reanalyze the data reported in this paper is available from the lead contact upon request.

### Experimental model and study participant details

#### Cohort enrollment and data collection

This analysis is a non-interventional, retrospective study conducted with data obtained from TriNetX, LLC (“TriNetX”). TriNetX is a global federated health research network that provides access to electronic medical records (EMRs) from healthcare organizations (HCOs) worldwide. The analysis was conducted with the TriNetX Global Collaborative Network, which provides access to data containing diagnoses, procedures, medications, laboratory values, and genomic information from approximately 130 million patients from 107 HCOs from around the world (60 from the US, 24 from EMEA countries, 9 from Asia-Pacific countries and 14 from Latin America region). TriNetX data is updated periodically asynchronously and has been used to run multiple studies published in several peer-reviewed scientific journals. Research studies using TriNetX do not require ethical approval as a federated network. The identity of participating HCOs and their contributions to each dataset is kept confidential by ethical norms and regulatory frameworks that prevent data re-identification. The TriNetX platform only uses aggregated counts and statistical summaries of de-identified information. No Protected Health Information or Personal Data is made available to the platform users. All data collection, processing, and transmission were performed in compliance with all Data Protection laws applicable to the contributing HCOs, including the EU Data Protection Law Regulation 2016/679, the General Data Protection Regulation on the protection of natural persons concerning the processing of personal data and the Health Insurance Portability and Accountability Act (HIPAA) the US federal law which protects the privacy and security of healthcare data. The Global Collaborative Networks is a distributed network, and analytics are performed on anonymized or pseudonymized/de-identified (per HIPAA) data housed at the HCOs, with only aggregate results being returned to the TriNetX platform.^19^ Individual personal data does not leave the HCO. TriNetX is ISO 27001:2013 certified and maintains a robust IT security program that protects personal and health care data.

Patients included in the study were all female and were selected from the network Global Collaborative Network of TriNetX Platform with 96 healthcare organizations (HCOs). For the first cohort, a total of 33 providers responded. We define in TriNetX cohort patients with a diagnosis of endometriosis (ICD-10CM: N80) between 2005 and 2018, being older than 18 years, that did not meet the exclusion criteria: (1) having a diagnosis of cancer of the endometrium (ICD-10-CM: C54.1, ICD-O: C54.1) before the endometriosis or (2) not having a record of death or a follow-up visit at least one year after the first endometriosis diagnosis. The final cohort of patients with endometriosis included 197,196 patients who matched the above query criteria. The control cohort included 6,455,556 patients who matched the query criteria: females encountering health services for examinations (ICD-10-CM: Z00-Z13) with BMI data, without endometriosis and a previous diagnosis of malignant neoplasm of the endometrium. Demographic and clinical characteristics of patients, as well as race, were provided according to cohorts in Table 1. Information on ethnicity and ancestry was missing.

These two cohorts were used to calculate the relative risk of developing endometrial cancer (EC). The analysis also included the risk comparison stratified by histology (invasive endometroid adenocarcinoma, invasive serous and clear cell) and staging, for patients for which that information was available. A survival analysis was also executed among those patients who developed EC in both cohorts. This survival analysis was also performed with the subcohorts of patients treated with radiotherapy, chemotherapy, or both treatments (Tables S1 and S2).

### Methods details

#### Propensity score estimation and matching

Cohorts were propensity scored matched 1:1 for age, race, inflammatory diseases of female pelvic organs (ICD10CM: N70-N77), family history of primary malignant neoplasm (ICD10CM: Z80), malignant neoplasm of colon (ICD10CM: C18), malignant neoplasm of rectosigmoid junction (ICD10CM: C19), malignant neoplasm of rectum (ICD10CM: C20) and BMI.

### Quantification and statistical analysis

All analyses were generated with TriNetX platform software (TriNetX, Cambridge, MA) on 20th November 2023. The descriptive statistics of the baseline patient characteristics included absolute value (n), the relative incidence (%) for all the categorical variables, and a comparison between cohorts using Fisher’s exact test. Continuous variables were presented with mean value and standard deviation and compared with the t-test. After matching the endometriosis and the control cohort, Cox proportional hazard ratios (HR) were performed to compare the relative risk of developing EC. Data analysis was limited to 10 years after the control cohort’s endometriosis diagnosis or encounter visit. A Kaplan-Meier overall survival analysis was carried out among the patients from both groups that developed EC. Kaplan-Meier analysis was also performed on the subgroups of patients treated with chemotherapy, radiotherapy, or both treatments.
