# Supplementary material for: Endometriosis and endometrial cancer: A propensity score-adjusted real-world data study
Source: iScience. 2024 Apr 6;27(5):109680. doi: 10.1016/j.isci.2024.109680 (PMC11033158; doi:10.1016/j.isci.2024.109680)
Supplement: Document S1. Tables S1 and S2 [file mmc1.pdf]

iScience, Volume *27*

Supplemental information

Endometriosis and endometrial cancer: A propensity score-adjusted real-world data study

Alberto Farolfi, Nicola Gentili, Sara Testoni, Francesca Rusconi, Ilaria Massa, Valentina Danesi, Amelia Altavilla, Maria C. Cursano, Giorgia Gurioli, Salvatore L. Burgio, Gema Hernandez Ibarburu, and Ugo De Giorgi

Table S1: **List of codes used to define "chemotherapy" in TriNetX**

Code	Decription
NLM:VA:AN000	ANTINEOPLASTICS
NLM:ATC:L	ANTINEOPLASTIC AND IMMUNOMODULATING AGENTS
UMLS:CPT:1013438	Chemotherapy and Other Highly Complex Drug or Highly Complex Biologic Agent Administration
UMLS:SNOMED:367336001	Chemotherapy
UMLS:ICD10PCS:3E03305	Introduction of Other Antineoplastic into Peripheral Vein, Percutaneous Approach
UMLS:ICD10PCS:3E04305	Introduction of Other Antineoplastic into Central Vein, Percutaneous Approach
UMLS:ICD10CM:Z51.11	Encounter for antineoplastic chemotherapy
UMLS:ICD10PCS:3E00X29	Introduction of Other Anti-infective into Skin and Mucous Membranes, External Approach
UMLS:ICD10PCS:3E0R305	Introduction of Other Antineoplastic into Spinal Canal, Percutaneous Approach

Table S2: **List of codes used to define "radiotherapy" in TriNetX**

Code	Decription
NLM:CPT:1010843	Radiation Oncology Treatment
UMLS:SNOMED:108290001	Radiation oncology AND/OR radiotherapy
UMLS:ICD10PCS:D	Radiation Therapy, excluding D02* and DG2*
TNX:10010	Radiation (Tumor Registry)
UMLS:ICD10CM:Z51.0	Encounter for Antineoplastic Radiation Therapy
